# Liposuction in cancer-related lower extremity lymphedema: an investigative study on clinical applications

**DOI:** 10.1186/s12957-021-02472-3

**Published:** 2022-01-05

**Authors:** Jianfeng Xin, Yuguang Sun, Song Xia, Kun Chang, Chao Dong, Zhong Liu, Jian Dong, Wenbin Shen

**Affiliations:** 1 Department of Lymphatic Surgery, Beijing Shijitan Hospital, Capital Medical University, No. 10 Tieyi Rd., Haidian District, Beijing, 100038 China; 2 Department of Ultrasound, Sanfine International Hospital, Beijing, China; 3 Department of Radiology, Beijing Shijitan Hospital, Capital Medical University, Beijing, China

**Keywords:** Lymphedema, Lower extremity, Liposuction, Cancer

## Abstract

**Background:**

Lymphedema is a progressive, noncurable condition consisting of increases in subcutaneous fat and interstitial fluid in the limbs and fibrosis during later stages. The disease most commonly affects the limbs following injury to or removal of the lymph nodes. The aim of this study was to investigate the therapeutic outcomes of liposuction for cancer-related lower extremity lymphedema.

**Methods:**

Sixty-two patients with cancer-related lymphedema in the unilateral lower extremity were recruited for this study, and all patients underwent liposuction. The volume of hemorrhage and lipids, the operation time, and the volume changes of the affected extremity were compared by applying the *t* tests, and the subjective feelings of patients were compared with the chi-square tests.

**Results:**

The total lipid volume was 2539 ± 1253.5 ml, and the hemorrhage volume was 828 ± 311.8 ml. For the comparison of objective indices, (1) the percent volume differences (PVDs) before surgery, intraoperatively, and at the 3-month follow-up were 5.5 ± 12.2 vs. 11.6 ± 18.4 vs. 43.2 ± 23.7, *P* < 0.05, respectively; (2) greater lipid volumes and higher liposuction rates were observed for female patients, as was a smaller volume of hemorrhage; (3) greater hemorrhage volumes were observed in patients with a history of recurrent erysipelas; and (4) greater lipid volumes and liposuction rates (LRs) and smaller hemorrhage volumes were observed for stage II than for stage III patients.

**Conclusions:**

Liposuction is an effective therapy for cancer-related lower extremity lymphedema. Sex, stage, and recurrent erysipelas history influence the course and effect of liposuction.

## Introduction

Pelvic tumor surgery is traditionally characterized by several major complications, including secondary lower extremity lymphedema [1]. Cancer-related lower extremity lymphedema is associated with treatment modalities such as cancer surgery and radiation therapy, which can injure or remove the lymph nodes [2, 3]. Lymphedema is a disabling condition whereby damage to the lymphatic channels causes the accumulation of protein-rich lymphatic fluid in subcutaneous tissues, which can cause abnormal proliferation of subcutaneous adipose tissue [4, 5]. Liposuction is a surgical technique that uses various types of equipment to aspirate excess subcutaneous fat through small incisions made in the skin to improve body esthetics, and pressure garments are worn for 3 to 6 months after liposuction [6]. Since Illouz first reported the negative pressure suction method for fat aspiration in 1983, liposuction has been widely applied in various therapeutic fields [7]. The most common areas for fat removal are the abdomen, flanks, trochanteric region, lumbar region, gluteal region, thighs, and calves [8]. The therapeutic effect of liposuction for lymphedema is still controversial. Brorson reported that liposuction combined with postoperative controlled compression therapy (CCT) is effective for the treatment of limb lymphedema [9]. Chen et al. and Chollet et al. found that in liposuction without concurrent skin excision, the skin often does not sufficiently retract and can lead to wound healing complications [10, 11]. However, the research results of Qi showed that limb volume reduction was noted in all cases immediately after the operation, but this state of volume reduction remained at 3 to 12 months with a mean of 6.01 months, when low-stretch bandages or compression stockings were applied. Subsequently, the volume returned to the preoperative level [12]. Other researchers have found that liposuction can significantly reduce lymphedema limb volume, but excess extracellular fluid persists [13]. In addition, the potential surgical risks and the effects in different lymphedema patient groups have not yet been investigated.

To investigate the therapeutic effects of liposuction, the volume of aspirated fat (VAF) and the volume of blood loss (VBL) during the operation in different patient groups with cancer-related lower extremity lymphedema were evaluated. The volume difference (VD) before, during, and 3 months after surgeries was assessed.

## Materials and methods

### Study population

Patients with lymphedema admitted to Beijing Shijitan Hospital from January to June 2017 were reviewed, and the inclusion criteria were as follows: (1) only unilateral lower extremity secondary lymphedema was present; (2) according to the lymphedema staging established by the International Society of Lymphology (ISL) in 2013 [14], patients were classified as either stage II (spontaneously irreversible lymphedema) or stage III (lymphostatic elephantiasis); (3) the initial operation was liposuction; (4) compression stockings were worn postoperatively; and (5) patients were followed up for more than 3 months. The exclusion criteria were as follows: (1) patients with a history of surgical treatment for lymphedema and (2) lower extremity lymphedema treated by liposuction and other surgical methods simultaneously. This retrospective study was conducted after receiving approval from the Institutional Review Board of Beijing Shijitan Hospital, and a waiver of consent was obtained.

### Surgical technique

The operation was performed under general anesthesia with endotracheal intubation. A tumescent solution was infused into the subcutaneous adipose tissue. Liposuction equipment (GZXZ Resonance Liposuction System, YangguangZhongtian Medical Equipment Co., Ltd., Shanxi) was used to remove subcutaneous adipose and interstitial fluid. At the end of the operation, a Jackson-Pratt (JP) drain was placed subcutaneously. Then, the operated limb was wrapped in compression bandages. On postoperative day 3, after a dressing change and drain removal, a compression garment was applied and used continuously. The patient was allowed to perform rehabilitation exercises.

### Measurements

- *Body mass index (BMI)*: BMI = weight (kg)/height^2^ (m^2^)
- *VD* and *PVD*: The VD and PVD were based on limb measurements and the volume calculation method devised by Brorson and Hoijer [15].
  $$\mathrm{VD}=\mathrm{affected}\ \mathrm{limb}\ \mathrm{volume}-\mathrm{healthy}\ \mathrm{limb}\ \mathrm{volume}$$

$$\mathrm{PVD}=\left[\left(\mathrm{affected}\ \mathrm{limb}\ \mathrm{volume}-\mathrm{healthy}\ \mathrm{limb}\ \mathrm{volume}\right)/\mathrm{healthy}\ \mathrm{limb}\ \mathrm{volume}\right]\times 100\%$$

- *VBL*: Preoperative hemoglobin, postoperative hemoglobin, and the intraoperative blood transfusion volume were recorded. Preoperative blood volume was obtained based on the formula derived by Nadler et al. [16]. Subsequently, the volume of blood loss was calculated according to the formula derived by Budny et al. [17].
  $$\mathrm{VBL}\ \left(\mathrm{ml}\right)=\mathrm{preoperative}\ \mathrm{blood}\ \mathrm{volume}\times \left[\left(\mathrm{preoperative}\ \mathrm{hemoglobin}-\mathrm{postoperative}\ \mathrm{hemoglobin}\right)\div \mathrm{preoperative}\ \mathrm{hemoglobin}\right]+\mathrm{volume}\ \mathrm{of}\ \mathrm{intraoperative}\ \mathrm{blood}\kern0.5em \mathrm{transfusion}$$
- *Liposuction rate (LR)*: LR = VAF/duration of operation.

### Postoperative follow-up

Limb shaping was completed 3 to 6 months postoperatively [18], so the follow-up was carried out 3 months after the operation. Patient follow-up consisted of measuring BMI and PVD. Patients completed a questionnaire to evaluate their subjective feelings, including stiffness, tension, heaviness, tenderness, pain, numbness, fatigue, and weakness.

### Statistical analysis

Data are presented as the mean ± standard deviation (mean ± SD). The Kolmogorov-Smirnov test was performed to verify that the parameters were normally distributed. The means of the two samples were compared by applying a *t* test of independent samples. The PVDs of the samples were compared by applying a *t* test of paired samples. A corrected *t* test was employed when there was a lack of variance. The chi-square was utilized to test the rate. *P* < 0.05 was considered statistically significant. The data were analyzed using SPSS version 20.0 (IBM, Armonk, NY, USA).

## Results

### Patient characteristics

A total of 62 patients were enrolled, including 16 males and 46 females, whose mean age was 55.6 ± 11.3 years (range 21–75). The mean duration of the disease was 55.8 months (range 12–288). Twenty-nine cases were in stage II (swelling not improved by limb elevation; with or without pitting edema), and 33 cases were in stage III (lymphedema is characterized by significant nonpitting swelling, fibroadipose deposition, hyperkeratosis, and acanthosis). Thirty-one patients presented with left lower extremity lymphedema, and 31 patients presented with right lower extremity lymphedema. Twenty patients had a history of recurrent erysipelas. The causes of lymphedema included lymphedema secondary to cervical cancer in 29 cases, endometrial cancer in 11 cases, inguinal stromal tumor in 8 cases, ovarian cancer in 3 cases, testicular cancer in 3 cases, lymphoma in 3 cases, rectal cancer in 2 cases, penile cancer in 2 cases, and bladder cancer in 1 case.

### Surgical outcomes

**Fig. 1:**
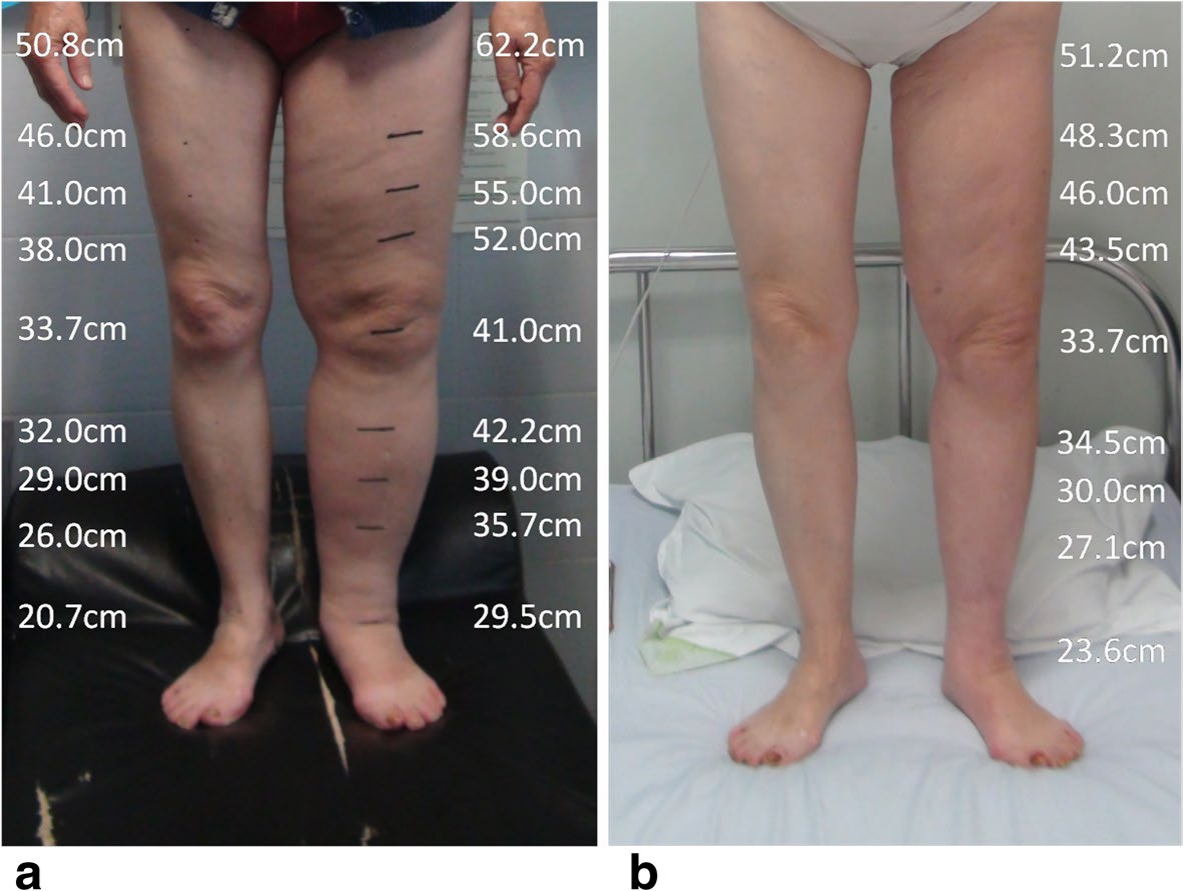
The patient, a 67-year-old female, had secondary lymphedema of the left lower extremity. **a** Preoperation. **b** Three months after the operation

**Fig. 2:**
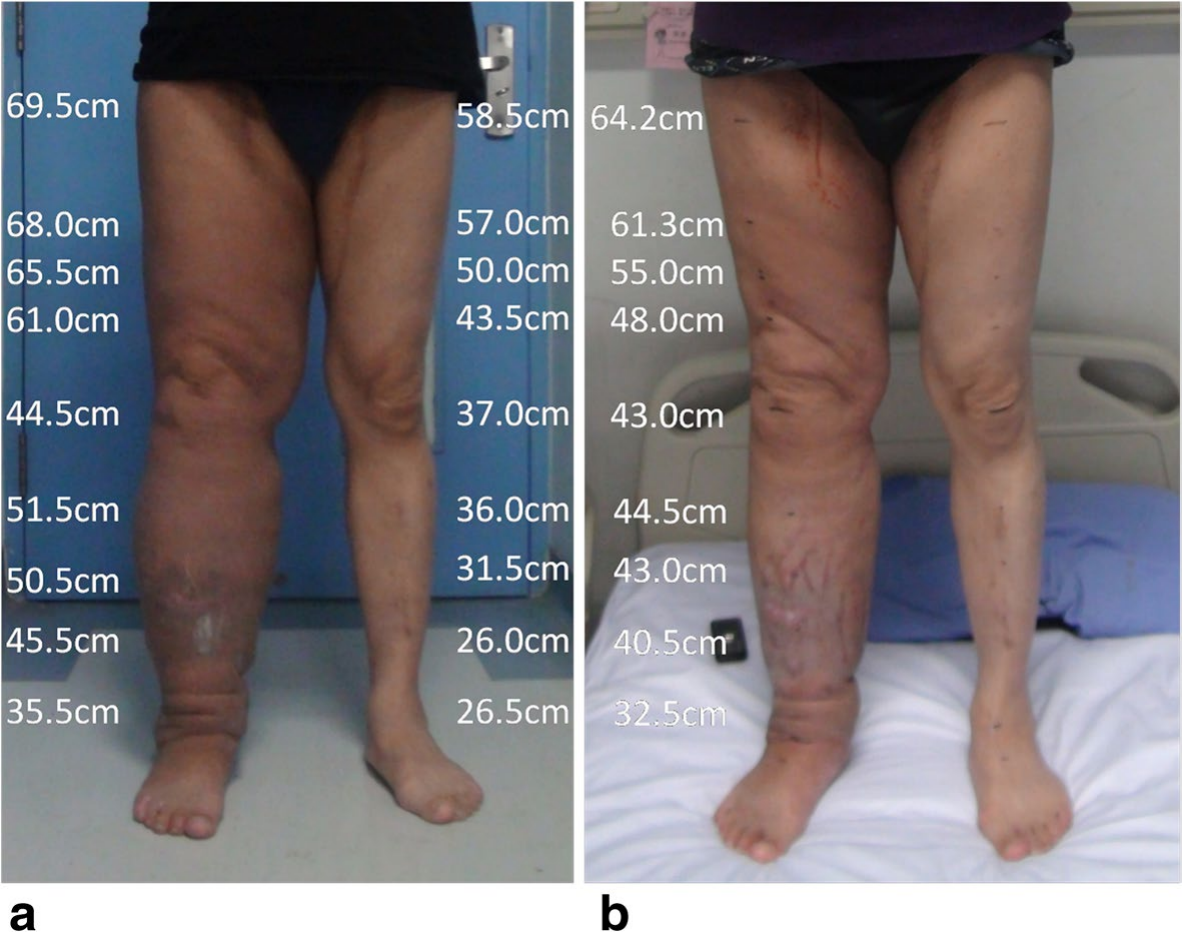
The patient, a 55-year-old male, had secondary lymphedema of the right lower extremity. **a** Preoperation. **b** Three months after the operation

In this study, 62 patients successfully underwent surgery without skin necrosis, subcutaneous effusion, infection, or other complications (Figs. 1b and 2b). The operation time was 176 ± 44.9 min (range 110–300). The VAF was 2539 ± 1253.5ml (range 400–5500). The estimated blood loss (EBL) was 828 ± 311.8 ml (range 224–1585). Intraoperative homologous blood transfusion was performed in 3 patients. The LR was 14.8 ± 7.7 ml/min (range 1.0–30.0). The appearance of the lymphedematous extremity significantly improved by 3 months postoperatively. The preoperative BMI was 26.7 ± 4.2, and the postoperative 3-month BMI was 25.9 ± 4.0. The difference was not statistically significant (*t* = 1.038, *P* = 0.301).

The preoperative, postoperative, and 3-month follow-up PVDs were 43.2 ± 23.7%, 5.5 ± 12.2%, and 11.6 ± 18.4%, respectively. The PVD at the postoperative and 3-month follow-ups significantly had decreased compared with that preoperatively (*P* < 0.05), but it significantly increased at the 3-month follow-up compared with that postoperation (*P* < 0.05).

The VAF and LR were higher in females than in males, whereas the EBL and PVD at the preoperative, postoperative, and 3-month follow-ups were greater in males than in females (Table 1). The EBL was higher in the recurrent erysipelas group than in the nonerysipelas group (Table 2). The VAF and LR were higher in the stage II group than in the stage III group. In contrast, the EBL and PVD preoperatively, postoperatively, and at the 3-month follow-up were lower in the stage II group (Table 3).

**Table 1:** Comparison of surgical results between male and female patients with secondary lymphedema of the lower extremities (mean ± SD)

	Male	Female	*t*	*P* value
*N*	16	46	–	–
VBL (ml)	986 ± 346	774 ± 282	2.434	0.018
VAF (ml)	1659 ± 1223	2845 ± 1122	− 3.559	0.001
LR (ml/min)	8.8 ± 5.9	16.9 ± 7.2	− 4.013	< 0.001
PVD				
Preoperation (%)	56.8 ± 22.1	38.4 ± 22.6	2.807	0.007
Postoperation (%)	16.5 ± 13.7	1.7 ± 9.1	4.034	0.001
3-month follow-up (%)	22.9 ± 18.6	7.7 ± 16.9	3.024	0.004

*N* numbers, *VBL* volume of blood loss, *VAF* volume of aspirated fat, *LR* liposuction rate, *PVD* percent volume difference

**Table 2:** Comparison of surgical results between the recurrent erysipelas and the nonerysipelas group with secondary lymphedema of the lower extremities (mean ± SD)

	Recurrent erysipelas	Nonerysipelas	*t*	*P* value
*N*	20	42	–	–
VBL (ml)	1000 ± 315	747 ± 278	3.201	0.002
VAF (ml)	2982 ± 1412	2328 ± 1127	1.965	0.054
LR (ml/min)	16.3 ± 7.7	14.1 ± 7.7	1.057	0.295
PVD				
Preoperation (%)	49.0 ± 27.5	40.4 ± 21.5	1.343	0.184
Postoperation (%)	7.3 ± 13.7	4.6 ± 11.5	0.808	0.422
3-month follow-up (%)	14.9 ± 17.9	10.1 ± 18.7	0.951	0.346

*N* numbers, *VBL* volume of blood loss, *VAF* volume of aspirated fat, *LR* liposuction rate, *PVD* percent volume difference

**Table 3:** Comparison of surgical results between the stage II and stage III groups with secondary lymphedema of the lower extremities (mean ± SD)

	Stage II	Stage III	*t*	*P* value
*N*	29	33	–	–
VBL (ml)	689 ± 249	950 ± 313	− 3.598	0.001
VAF (ml)	2875 ± 1247	2243 ± 1199	2.031	0.047
LR (ml/min)	18.0 ± 8.4	12.1 ± 6.0	3.189	0.002
PVD				
Preoperation (%)	28.1 ± 18.4	56.5 ± 19.7	− 5.821	< 0.001
Postoperation (%)	-3.2 ± 6.2	13.3 ± 10.9	− 7.438	< 0.001
3-month follow-up (%)	1.7 ± 15.3	20.4 ± 16.6	− 4.576	< 0.001

*N* numbers, *VBL* volume of blood loss, *VAF* volume of aspirated fat, *LR* liposuction rate, *PVD* percent volume difference

The feeling of heaviness and fatigue in the operated limb was alleviated by the 3-month follow-up compared with that preoperatively, whereas feelings of stiffness, tenderness, and tightness worsened. There were no significant differences in pain, numbness, or weakness between preoperative and 3-month follow-ups (Table 4).

**Table 4:** Comparison of subjective sensation between the preoperative visit and the 3-month follow-up in 62 patients with secondary lymphedema of the lower extremity [case (%)]

	Preoperation	3-month follow-up	*χ*^2^	*P* value
Stiffness	25 (40.3)	36 (58.1)	3.904	0.048
Tightness	19 (30.6)	35 (56.4)	8.398	0.004
Heaviness	49 (80.6)	34 (54.8)	9.448	0.002
Tenderness	5 (7.9)	13 (21.0)	4.305	0.038
Pain	6 (9.7)	13 (21.0)	3.046	0.081
Numbness	33 (53.2)	41 (66.1)	2.145	0.143
Fatigue	44 (71.0)	27 (43.5)	9.523	0.002
Weakness	7 (11.3)	11 (17.7)	1.040	0.308

## Discussion

Worldwide, secondary lymphedema most commonly occurs following cancer treatment [19]. A variety of conservative therapies have been reported, including complete decongestion therapy (CDT) [20, 21], as well as microsurgical reconstruction, including lymphatic venous anastomosis [22] and lymph node transplantation [23, 24]. Failure of these treatments to provide a complete reduction in patients with long-standing pronounced lymphedema is due to the persistence of excess newly formed subcutaneous adipose tissue in response to slow or absent lymph flow that is not removed in patients with chronic lymphedema. Liposuction has drawn increased attention for surgical treatment of lymphedema because it can remove hypertrophied adipose tissue.

Here, we have demonstrated that liposuction surgery improved both quality of life and volumetric measurements in patients with lymphedema. Overall, the PVD was significantly lower postoperatively and at the 3-month follow-up than preoperatively. The mean estimated blood loss (EBL) was 828 ml, which was much greater than the EBL of 292 ml when liposuction is performed in obese patients [25]. The reason may be that there is abnormal hyperplasia of subcutaneous adipose tissue in lymphedematous limbs and that the proliferating adipose layer contains less regular fascial septae, which poses more difficulty for liposuction of lymphedematous limbs, aggravates peripheral vascular injury, and increases bleeding compared with conventional liposuction. Therefore, patients with anemia should be treated proactively before liposuction. Some patients may need packed red blood cells (pRBCs) before surgery. In this study, 3 patients were given intraoperative homologous blood transfusions.

Males had a significantly higher PVD than females preoperatively, postoperatively, and at the 3-month follow-up but a significantly lower VAF and greater EBL. This suggests that there may be differences between males and females in the occurrence and development of lymphedema. Perhaps the abnormality of the lymphedematous lower extremity in males is greater than that in females. In addition, subcutaneous tissue hyperplasia and fibrosis may be aggravated as the disease progresses. We also found that the LR in males was significantly lower than that in females during surgery. That is, it was more difficult to extract fat in males. Moreover, the effect of different hormone levels in males and females on the composition of subcutaneous tissue [26, 27], as well as the poor compliance among males, may account for the aforementioned differences, which need to be further investigated.

Lymphedema is one of the risk factors for the occurrence of erysipelas [28]. Recurrent erysipelas can lead to subcutaneous tissue fibrosis, which can in turn aggravate lymphedema. In a study by Kosenkov et al., a correlation was found between the occurrence of erysipelas and the degree of lymphedema, and they aggravated each other [29]. In our study, the recurrent erysipelas group was noted to have a greater EBL than the nonerysipelas group. This suggests that patients with recurrent erysipelas may have more fibrosis in the subcutaneous tissue, which could increase the difficulty and risk of the surgery.

The PVD of the stage III group was significantly larger than that of the stage II group preoperatively, postoperatively, and at the 3-month follow-up, whereas the VAF and LR of the stage III group were significantly lower than those of the stage II group. In addition, the EBL of the stage III group was greater than that of the stage II group. These results suggest that the longer the disease period, the more difficult liposuction may become, and the more blood loss may ensue. It can be deduced that the surgical outcome may also be worse. The reason may be that the degree of fibrosis in subcutaneous tissue gradually increases over the course of the disease. Sun et al. also believe that skin hardness is positively correlated with the stage of lymphedema [30]. Consequently, the outcome of liposuction and volume reduction in advanced limb lymphedema may not be as good as those in early lymphedema.

Regarding the subjective feelings of the patients, the feelings of heaviness and fatigue in the affected limbs were significantly reduced at the 3-month follow-up compared with the preoperative values, but the feelings of stiffness, tenderness, and tightness of the lymphedematous limb were more severe than the preoperative values. This may be related to injury to subcutaneous nerves and inflammatory reactions after liposuction. It is worth noting that 58.1% of patients still felt stiff, 56.4% felt tight, and 54.8% felt heavy 3 months after liposuction. The reason may be that compression stockings fail to effectively improve lymphatic reflux despite the significantly reduced volume of the lymphedematous limb after liposuction.

The data also show that the PVD increases significantly at the 3-month follow-up after liposuction, which is consistent with the subjective sensory changes of the patients. Qi et al. compared the outcome of liposuction in 17 patients (7 with upper limb lymphedema and 10 with lower limb lymphedema). Substantial limb volume reduction was noted in all cases immediately after the operation. This state of volume reduction remained at 3 to 12 months, with a mean of 6.01 months, when low-stretch bandages or compression stockings were applied. Subsequently, the volume returned to the preoperative level [12]. This implies that liposuction combined with more effective postoperative measures to ameliorate the reflux of the lymph could contribute to better surgical outcomes for extremity lymphedema.

## Conclusions

Liposuction has positive effects on the treatment of cancer-related lower extremity lymphedema. Nevertheless, compared with traditional liposuction in plastic surgery, it is riskier and more difficult. Moreover, sex differences, history of erysipelas, and disease stage should be taken into account during the treatment process, especially during the perioperative period.

## Data Availability

Not applicable.

## Abbreviations

EBL: Estimated blood loss
CCT: Controlled compression therapy
VBL: Volume of blood loss
VAF: Volume of aspirated fat
LR: Liposuction rate
PVD: Percent volume difference
BMI: Body mass index
