# Correction to: Liposuction in cancer-related lower extremity lymphedema: an investigative study on clinical applications

**DOI:** 10.1186/s12957-022-02522-4

**Published:** 2022-02-24

**Authors:** Jianfeng Xin, Yuguang Sun, Song Xia, Kun Chang, Chao Dong, Zhong Liu, Jian Dong, Wenbin Shen

**Affiliations:** 1 Department of Lymphatic Surgery, Beijing Shijitan Hospital, Capital Medical University, No. 10 Tieyi Rd., Haidian District, Beijing, 100038 China; 2 Department of Ultrasound, Sanfine International Hospital, Beijing, China; 3 Department of Radiology, Beijing Shijitan Hospital, Capital Medical University, Beijing, China

**Correction to: World J Surg Oncol 20, 1-7 (2022)**

**https://doi.org/10.1186/s12957-021-02472-3**

Following the publication of the original article [1], the author reported that the correct corresponding email address “shenwenbin@bjsjth.cn” should be “shenwb@bjsjth.cn”.
